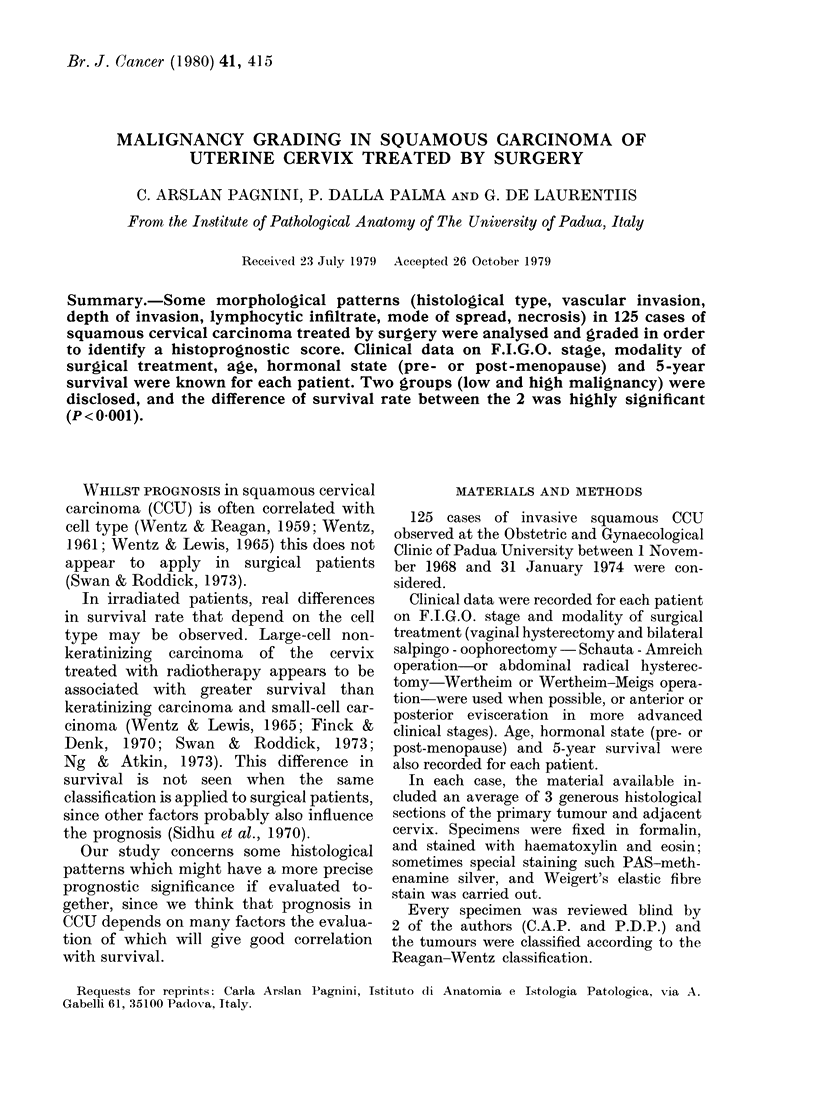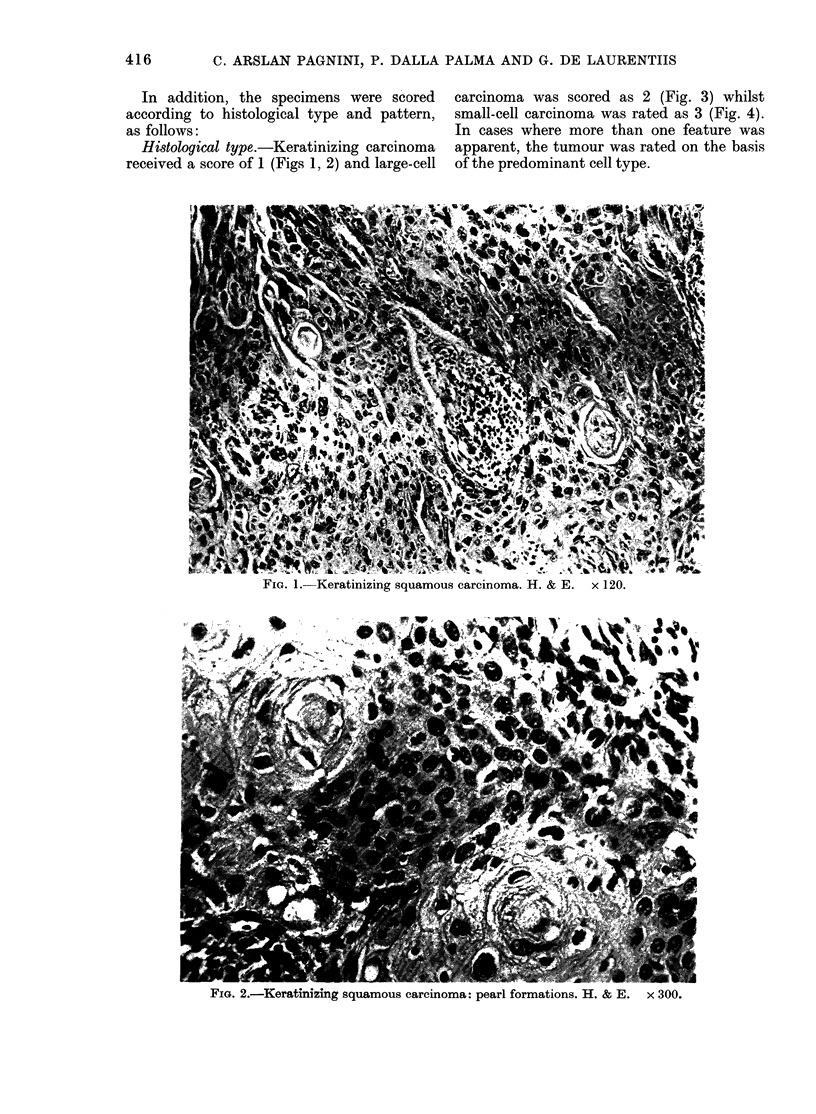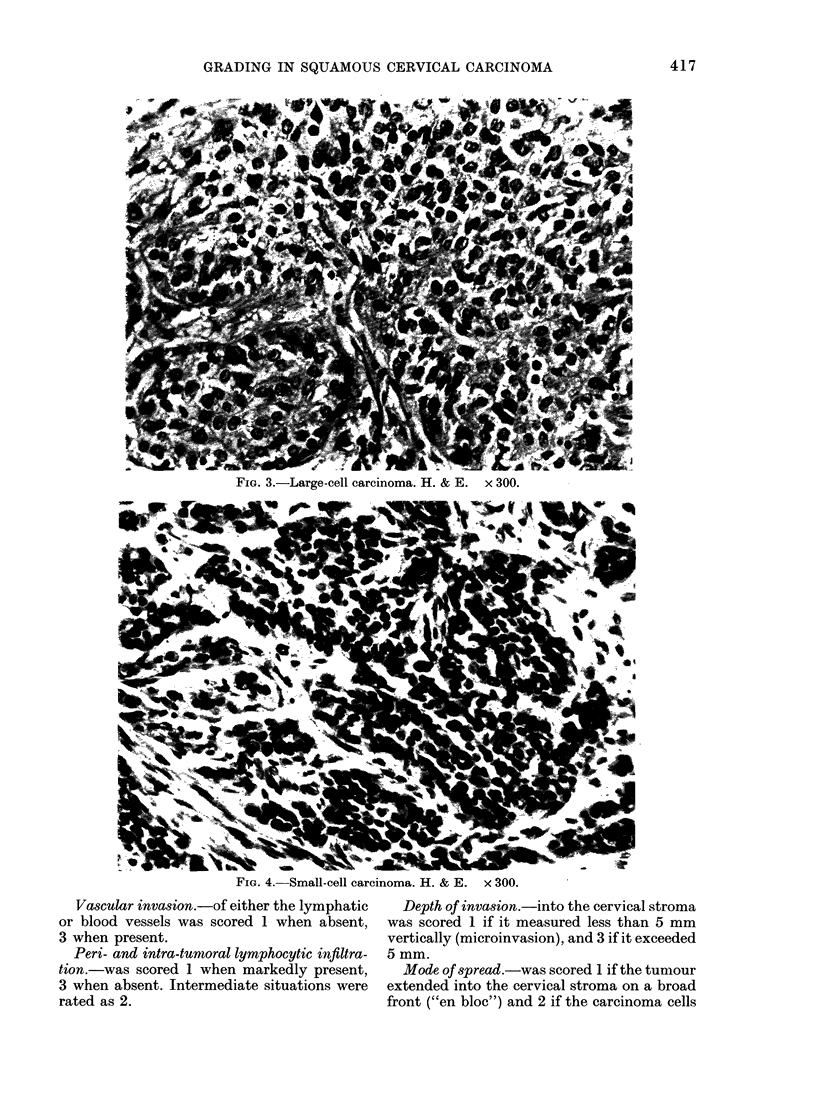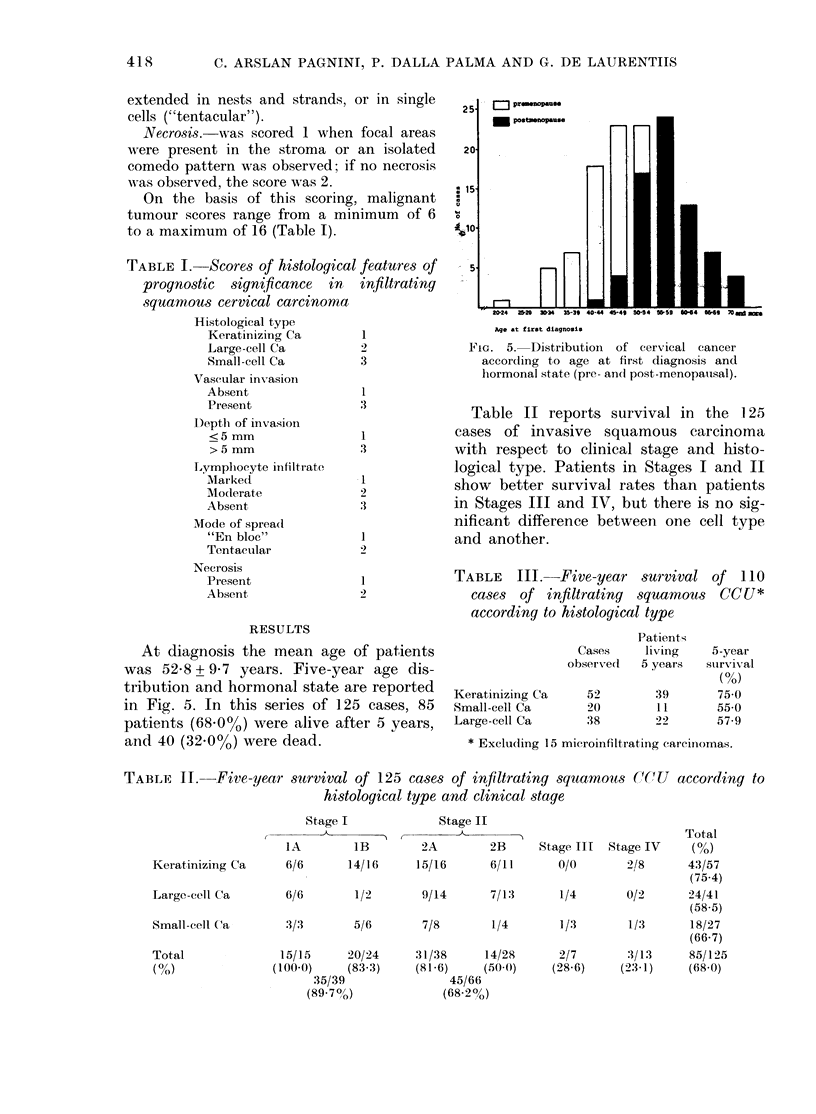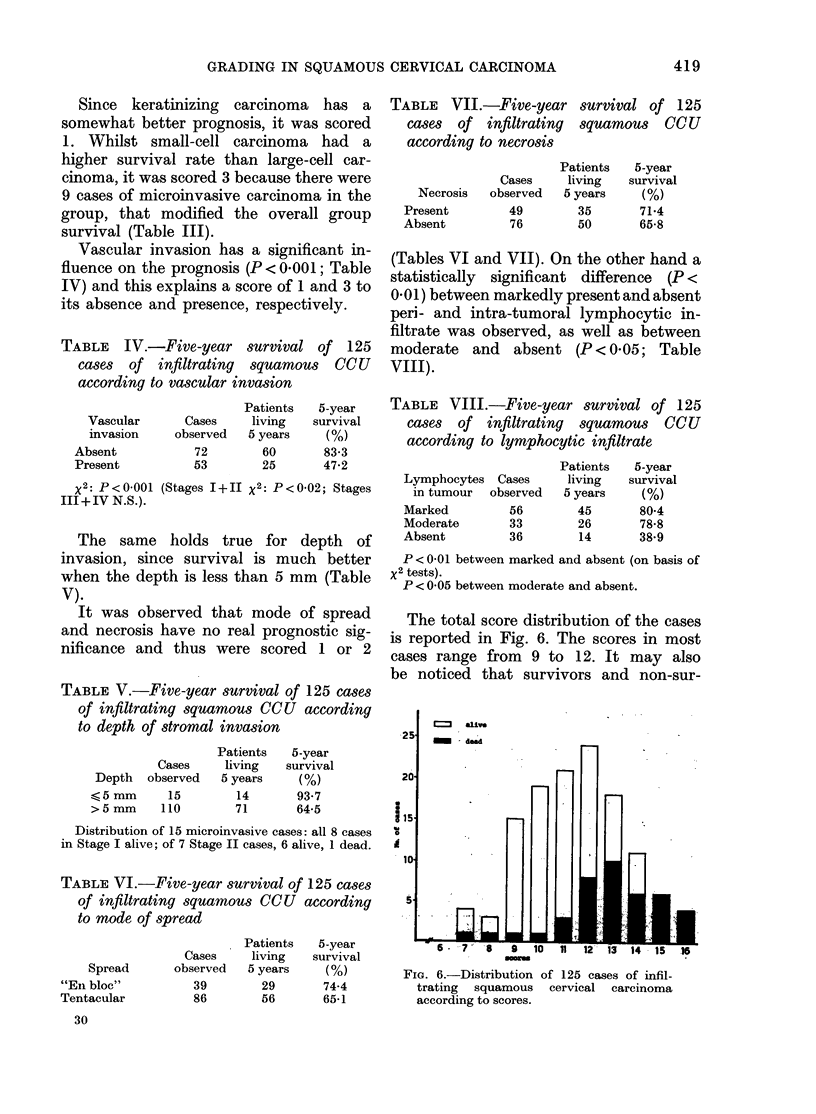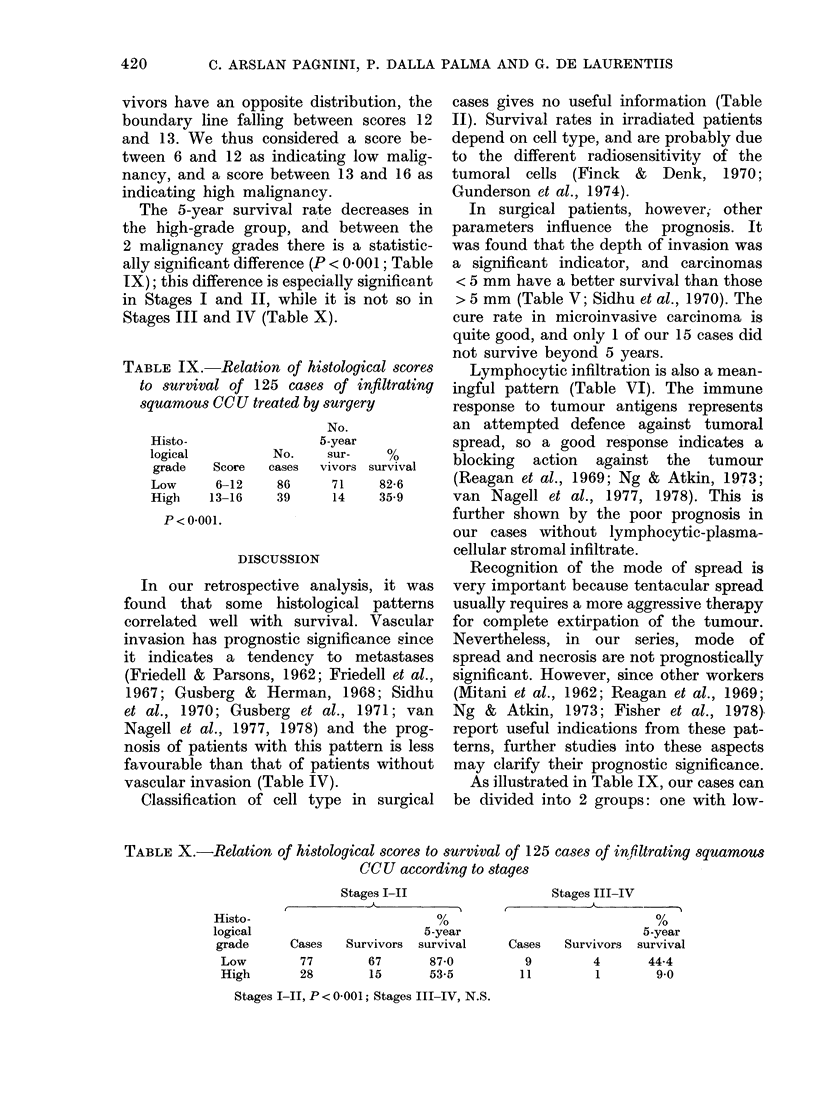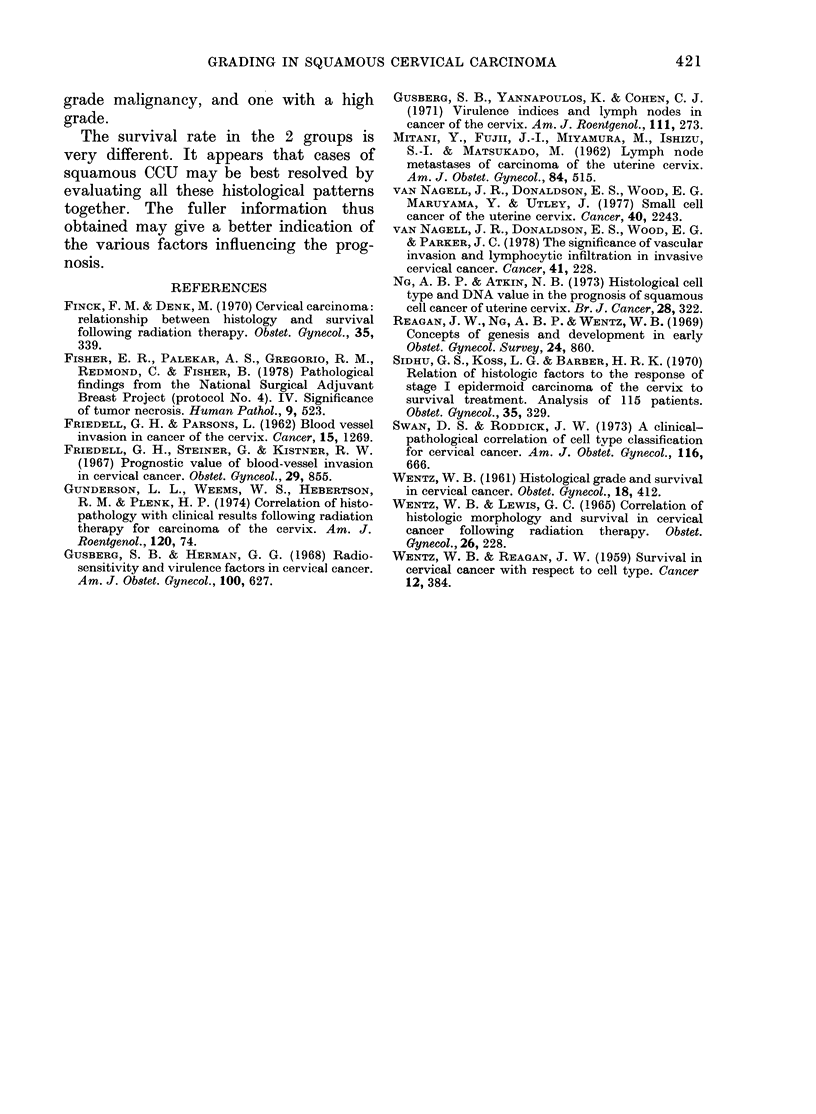# Malignancy grading in squamous carcinoma of uterine cervix treated by surgery.

**DOI:** 10.1038/bjc.1980.65

**Published:** 1980-03

**Authors:** C. A. Pagnini, P. Della Palma, G. De Laurentiis

## Abstract

**Images:**


*Br. J. Cancer* (1980) **41**, 415

### MALIGNANCY GRADING IN SQUAMOUS CARCINOMA OF UTERINE CERVIX TREATED BY SURGERY

C. ARSLAN PAGNINI, P. DALLA PALMA AND G. DE LAURENTIIS

*From the Institute of Pathological Anatomy of The University of Padua, Italy*

Received 23 July 1979  Accepted 26 October 1979

**Summary.—Some morphological patterns (histological type, vascular invasion, depth of invasion, lymphocytic infiltrate, mode of spread, necrosis) in 125 cases of squamous cervical carcinoma treated by surgery were analysed and graded in order to identify a histoprognostic score. Clinical data on F.I.G.O. stage, modality of surgical treatment, age, hormonal state (pre- or post-menopause) and 5-year survival were known for each patient. Two groups (low and high malignancy) were disclosed, and the difference of survival rate between the 2 was highly significant ($P < 0{\cdot}001$).**

WHILST PROGNOSIS in squamous cervical carcinoma (CCU) is often correlated with cell type (Wentz & Reagan, 1959; Wentz, 1961; Wentz & Lewis, 1965) this does not appear to apply in surgical patients (Swan & Roddick, 1973).

In irradiated patients, real differences in survival rate that depend on the cell type may be observed. Large-cell non-keratinizing carcinoma of the cervix treated with radiotherapy appears to be associated with greater survival than keratinizing carcinoma and small-cell carcinoma (Wentz & Lewis, 1965; Finck & Denk, 1970; Swan & Roddick, 1973; Ng & Atkin, 1973). This difference in survival is not seen when the same classification is applied to surgical patients, since other factors probably also influence the prognosis (Sidhu *et al.*, 1970).

Our study concerns some histological patterns which might have a more precise prognostic significance if evaluated together, since we think that prognosis in CCU depends on many factors the evaluation of which will give good correlation with survival.

### MATERIALS AND METHODS

125 cases of invasive squamous CCU observed at the Obstetric and Gynaecological Clinic of Padua University between 1 November 1968 and 31 January 1974 were considered.

Clinical data were recorded for each patient on F.I.G.O. stage and modality of surgical treatment (vaginal hysterectomy and bilateral salpingo - oophorectomy — Schauta - Amreich operation—or abdominal radical hysterectomy—Wertheim or Wertheim–Meigs operation—were used when possible, or anterior or posterior evisceration in more advanced clinical stages). Age, hormonal state (pre- or post-menopause) and 5-year survival were also recorded for each patient.

In each case, the material available included an average of 3 generous histological sections of the primary tumour and adjacent cervix. Specimens were fixed in formalin, and stained with haematoxylin and eosin; sometimes special staining such PAS–methenamine silver, and Weigert's elastic fibre stain was carried out.

Every specimen was reviewed blind by 2 of the authors (C.A.P. and P.D.P.) and the tumours were classified according to the Reagan–Wentz classification.

Requests for reprints: Carla Arslan Pagnini, Istituto di Anatomia e Istologia Patologica, via A. Gabelli 61, 35100 Padova, Italy.

In addition, the specimens were scored according to histological type and pattern, as follows:

*Histological type.*—Keratinizing carcinoma received a score of 1 (Figs 1, 2) and large-cell carcinoma was scored as 2 (Fig. 3) whilst small-cell carcinoma was rated as 3 (Fig. 4). In cases where more than one feature was apparent, the tumour was rated on the basis of the predominant cell type.

FIG. 1.—Keratinizing squamous carcinoma. H. & E. ×120.

FIG. 2.—Keratinizing squamous carcinoma: pearl formations. H. & E. ×300.

FIG. 3.—Large-cell carcinoma. H. & E. ×300.

FIG. 4.—Small-cell carcinoma. H. & E. ×300.

*Vascular invasion.*—of either the lymphatic or blood vessels was scored 1 when absent, 3 when present.

*Peri- and intra-tumoral lymphocytic infiltration.*—was scored 1 when markedly present, 3 when absent. Intermediate situations were rated as 2.

*Depth of invasion.*—into the cervical stroma was scored 1 if it measured less than 5 mm vertically (microinvasion), and 3 if it exceeded 5 mm.

*Mode of spread.*—was scored 1 if the tumour extended into the cervical stroma on a broad front ("en bloc") and 2 if the carcinoma cells extended in nests and strands, or in single cells ("tentacular").

*Necrosis.*—was scored 1 when focal areas were present in the stroma or an isolated comedo pattern was observed; if no necrosis was observed, the score was 2.

On the basis of this scoring, malignant tumour scores range from a minimum of 6 to a maximum of 16 (Table I).

TABLE I.—*Scores of histological features of prognostic significance in infiltrating squamous cervical carcinoma*

| | |
|---|---|
| Histological type | |
| Keratinizing Ca | 1 |
| Large-cell Ca | 2 |
| Small-cell Ca | 3 |
| Vascular invasion | |
| Absent | 1 |
| Present | 3 |
| Depth of invasion | |
| ≤5 mm | 1 |
| >5 mm | 3 |
| Lymphocyte infiltrate | |
| Marked | 1 |
| Moderate | 2 |
| Absent | 3 |
| Mode of spread | |
| "En bloc" | 1 |
| Tentacular | 2 |
| Necrosis | |
| Present | 1 |
| Absent | 2 |

RESULTS

At diagnosis the mean age of patients was 52·8 ± 9·7 years. Five-year age distribution and hormonal state are reported in Fig. 5. In this series of 125 cases, 85 patients (68·0%) were alive after 5 years, and 40 (32·0%) were dead.

FIG. 5.—Distribution of cervical cancer according to age at first diagnosis and hormonal state (pre- and post-menopausal).

Table II reports survival in the 125 cases of invasive squamous carcinoma with respect to clinical stage and histological type. Patients in Stages I and II show better survival rates than patients in Stages III and IV, but there is no significant difference between one cell type and another.

TABLE III.—*Five-year survival of* 110 *cases of infiltrating squamous CCU* according to histological type*

| | Cases observed | Patients living 5 years | 5-year survival (%) |
|---|---|---|---|
| Keratinizing Ca | 52 | 39 | 75·0 |
| Small-cell Ca | 20 | 11 | 55·0 |
| Large-cell Ca | 38 | 22 | 57·9 |

* Excluding 15 microinfiltrating carcinomas.

TABLE II.—*Five-year survival of* 125 *cases of infiltrating squamous CCU according to histological type and clinical stage*

| | Stage I | | Stage II | | | | |
|---|---|---|---|---|---|---|---|
| | 1A | 1B | 2A | 2B | Stage III | Stage IV | Total (%) |
| Keratinizing Ca | 6/6 | 14/16 | 15/16 | 6/11 | 0/0 | 2/8 | 43/57 (75·4) |
| Large-cell Ca | 6/6 | 1/2 | 9/14 | 7/13 | 1/4 | 0/2 | 24/41 (58·5) |
| Small-cell Ca | 3/3 | 5/6 | 7/8 | 1/4 | 1/3 | 1/3 | 18/27 (66·7) |
| Total (%) | 15/15 (100·0) | 20/24 (83·3) | 31/38 (81·6) | 14/28 (50·0) | 2/7 (28·6) | 3/13 (23·1) | 85/125 (68·0) |
| | 35/39 (89·7%) | | 45/66 (68·2%) | | | | |

Since keratinizing carcinoma has a somewhat better prognosis, it was scored 1. Whilst small-cell carcinoma had a higher survival rate than large-cell carcinoma, it was scored 3 because there were 9 cases of microinvasive carcinoma in the group, that modified the overall group survival (Table III).

Vascular invasion has a significant influence on the prognosis ($P < 0{\cdot}001$; Table IV) and this explains a score of 1 and 3 to its absence and presence, respectively.

TABLE IV.—*Five-year survival of* 125 *cases of infiltrating squamous CCU according to vascular invasion*

| Vascular invasion | Cases observed | Patients living 5 years | 5-year survival (%) |
|---|---|---|---|
| Absent | 72 | 60 | 83·3 |
| Present | 53 | 25 | 47·2 |

$\chi^2$: $P < 0{\cdot}001$ (Stages I+II $\chi^2$: $P < 0{\cdot}02$; Stages III+IV N.S.).

The same holds true for depth of invasion, since survival is much better when the depth is less than 5 mm (Table V).

It was observed that mode of spread and necrosis have no real prognostic significance and thus were scored 1 or 2 (Tables VI and VII). On the other hand a statistically significant difference ($P < 0{\cdot}01$) between markedly present and absent peri- and intra-tumoral lymphocytic infiltrate was observed, as well as between moderate and absent ($P < 0{\cdot}05$; Table VIII).

TABLE V.—*Five-year survival of* 125 *cases of infiltrating squamous CCU according to depth of stromal invasion*

| Depth | Cases observed | Patients living 5 years | 5-year survival (%) |
|---|---|---|---|
| ≤5 mm | 15 | 14 | 93·7 |
| >5 mm | 110 | 71 | 64·5 |

Distribution of 15 microinvasive cases: all 8 cases in Stage I alive; of 7 Stage II cases, 6 alive, 1 dead.

TABLE VI.—*Five-year survival of* 125 *cases of infiltrating squamous CCU according to mode of spread*

| Spread | Cases observed | Patients living 5 years | 5-year survival (%) |
|---|---|---|---|
| "En bloc" | 39 | 29 | 74·4 |
| Tentacular | 86 | 56 | 65·1 |

TABLE VII.—*Five-year survival of* 125 *cases of infiltrating squamous CCU according to necrosis*

| Necrosis | Cases observed | Patients living 5 years | 5-year survival (%) |
|---|---|---|---|
| Present | 49 | 35 | 71·4 |
| Absent | 76 | 50 | 65·8 |

TABLE VIII.—*Five-year survival of* 125 *cases of infiltrating squamous CCU according to lymphocytic infiltrate*

| Lymphocytes in tumour | Cases observed | Patients living 5 years | 5-year survival (%) |
|---|---|---|---|
| Marked | 56 | 45 | 80·4 |
| Moderate | 33 | 26 | 78·8 |
| Absent | 36 | 14 | 38·9 |

$P < 0{\cdot}01$ between marked and absent (on basis of $\chi^2$ tests).

$P < 0{\cdot}05$ between moderate and absent.

The total score distribution of the cases is reported in Fig. 6. The scores in most cases range from 9 to 12. It may also be noticed that survivors and non-sur-

FIG. 6.—Distribution of 125 cases of infiltrating squamous cervical carcinoma according to scores.

vivors have an opposite distribution, the boundary line falling between scores 12 and 13. We thus considered a score between 6 and 12 as indicating low malignancy, and a score between 13 and 16 as indicating high malignancy.

The 5-year survival rate decreases in the high-grade group, and between the 2 malignancy grades there is a statistically significant difference ($P < 0.001$; Table IX); this difference is especially significant in Stages I and II, while it is not so in Stages III and IV (Table X).

TABLE IX.—*Relation of histological scores to survival of* 125 *cases of infiltrating squamous CCU treated by surgery*

| Histological grade | Score | No. cases | No. 5-year survivors | % survival |
|---|---|---|---|---|
| Low | 6–12 | 86 | 71 | 82·6 |
| High | 13–16 | 39 | 14 | 35·9 |

$P < 0.001$.

### DISCUSSION

In our retrospective analysis, it was found that some histological patterns correlated well with survival. Vascular invasion has prognostic significance since it indicates a tendency to metastases (Friedell & Parsons, 1962; Friedell *et al.*, 1967; Gusberg & Herman, 1968; Sidhu *et al.*, 1970; Gusberg *et al.*, 1971; van Nagell *et al.*, 1977, 1978) and the prognosis of patients with this pattern is less favourable than that of patients without vascular invasion (Table IV).

Classification of cell type in surgical cases gives no useful information (Table II). Survival rates in irradiated patients depend on cell type, and are probably due to the different radiosensitivity of the tumoral cells (Finck & Denk, 1970; Gunderson *et al.*, 1974).

In surgical patients, however, other parameters influence the prognosis. It was found that the depth of invasion was a significant indicator, and carcinomas < 5 mm have a better survival than those > 5 mm (Table V; Sidhu *et al.*, 1970). The cure rate in microinvasive carcinoma is quite good, and only 1 of our 15 cases did not survive beyond 5 years.

Lymphocytic infiltration is also a meaningful pattern (Table VI). The immune response to tumour antigens represents an attempted defence against tumoral spread, so a good response indicates a blocking action against the tumour (Reagan *et al.*, 1969; Ng & Atkin, 1973; van Nagell *et al.*, 1977, 1978). This is further shown by the poor prognosis in our cases without lymphocytic-plasmacellular stromal infiltrate.

Recognition of the mode of spread is very important because tentacular spread usually requires a more aggressive therapy for complete extirpation of the tumour. Nevertheless, in our series, mode of spread and necrosis are not prognostically significant. However, since other workers (Mitani *et al.*, 1962; Reagan *et al.*, 1969; Ng & Atkin, 1973; Fisher *et al.*, 1978) report useful indications from these patterns, further studies into these aspects may clarify their prognostic significance.

As illustrated in Table IX, our cases can be divided into 2 groups: one with low-

TABLE X.—*Relation of histological scores to survival of* 125 *cases of infiltrating squamous CCU according to stages*

| Histological grade | Stages I–II Cases | Stages I–II Survivors | Stages I–II % 5-year survival | Stages III–IV Cases | Stages III–IV Survivors | Stages III–IV % 5-year survival |
|---|---|---|---|---|---|---|
| Low | 77 | 67 | 87·0 | 9 | 4 | 44·4 |
| High | 28 | 15 | 53·5 | 11 | 1 | 9·0 |

Stages I–II, $P < 0.001$; Stages III–IV, N.S.

grade malignancy, and one with a high grade.

The survival rate in the 2 groups is very different. It appears that cases of squamous CCU may be best resolved by evaluating all these histological patterns together. The fuller information thus obtained may give a better indication of the various factors influencing the prognosis.

### REFERENCES

FINCK, F. M. & DENK, M. (1970) Cervical carcinoma: relationship between histology and survival following radiation therapy. *Obstet. Gynecol.*, **35**, 339.

FISHER, E. R., PALEKAR, A. S., GREGORIO, R. M., REDMOND, C. & FISHER, B. (1978) Pathological findings from the National Surgical Adjuvant Breast Project (protocol No. 4). IV. Significance of tumor necrosis. *Human Pathol.*, **9**, 523.

FRIEDELL, G. H. & PARSONS, L. (1962) Blood vessel invasion in cancer of the cervix. *Cancer*, **15**, 1269.

FRIEDELL, G. H., STEINER, G. & KISTNER, R. W. (1967) Prognostic value of blood-vessel invasion in cervical cancer. *Obstet. Gynceol.*, **29**, 855.

GUNDERSON, L. L., WEEMS, W. S., HEBERTSON, R. M. & PLENK, H. P. (1974) Correlation of histopathology with clinical results following radiation therapy for carcinoma of the cervix. *Am. J. Roentgenol.*, **120**, 74.

GUSBERG, S. B. & HERMAN, G. G. (1968) Radiosensitivity and virulence factors in cervical cancer. *Am. J. Obstet. Gynecol.*, **100**, 627.

GUSBERG, S. B., YANNAPOULOS, K. & COHEN, C. J. (1971) Virulence indices and lymph nodes in cancer of the cervix. *Am. J. Roentgenol.*, **111**, 273.

MITANI, Y., FUJII, J.-I., MIYAMURA, M., ISHIZU, S.-I. & MATSUKADO, M. (1962) Lymph node metastases of carcinoma of the uterine cervix. *Am. J. Obstet. Gynecol.*, **84**, 515.

VAN NAGELL, J. R., DONALDSON, E. S., WOOD, E. G. MARUYAMA, Y. & UTLEY, J. (1977) Small cell cancer of the uterine cervix. *Cancer*, **40**, 2243.

VAN NAGELL, J. R., DONALDSON, E. S., WOOD, E. G. & PARKER, J. C. (1978) The significance of vascular invasion and lymphocytic infiltration in invasive cervical cancer. *Cancer*, **41**, 228.

NG, A. B. P. & ATKIN, N. B. (1973) Histological cell type and DNA value in the prognosis of squamous cell cancer of uterine cervix. *Br. J. Cancer*, **28**, 322.

REAGAN, J. W., NG, A. B. P. & WENTZ, W. B. (1969) Concepts of genesis and development in early *Obstet. Gynecol. Survey*, **24**, 860.

SIDHU, G. S., KOSS, L. G. & BARBER, H. R. K. (1970) Relation of histologic factors to the response of stage I epidermoid carcinoma of the cervix to survival treatment. Analysis of 115 patients. *Obstet. Gynecol.*, **35**, 329.

SWAN, D. S. & RODDICK, J. W. (1973) A clinical–pathological correlation of cell type classification for cervical cancer. *Am. J. Obstet. Gynecol.*, **116**, 666.

WENTZ, W. B. (1961) Histological grade and survival in cervical cancer. *Obstet. Gynecol.*, **18**, 412.

WENTZ, W. B. & LEWIS, G. C. (1965) Correlation of histologic morphology and survival in cervical cancer following radiation therapy. *Obstet. Gynecol.*, **26**, 228.

WENTZ, W. B. & REAGAN, J. W. (1959) Survival in cervical cancer with respect to cell type. *Cancer* **12**, 384.